# Retroperitoneoscopic donor nephrectomy with a gel-sealed hand-assist access device

**DOI:** 10.1186/1471-2490-13-7

**Published:** 2013-02-02

**Authors:** Kei Arai, Tsutomu Nishiyama, Noboru Hara, Takashi Kasahara, Kazuhide Saito, Kota Takahashi

**Affiliations:** 1 Division of Urology, Department of Regenerative and Transplant Medicine, Graduate School of Medical and Dental Sciences, Niigata University, Asahimachi 1, Niigata, 951-8510, Japan

**Keywords:** Donor nephrectomy, Retroperitoneoscopy, Gel-sealed access device

## Abstract

**Background:**

The hand-assisted technique enables the rapid extraction of the graft, shortening the warm ischemia time (WIT), and the retroperitoneoscopic approach is potentially associated with a less incidence of postoperative ileus in donor nephrectomy for living kidney transplantation. The aim of this study was to assess the efficacy and safety of retroperitoneoscopic donor nephrectomy with a gel-sealed hand-assist access device (GelPort), which is a wound sealing device that permits the access of the hand to the surgical field, free trocar site choice within it, and rapid conversion to open surgery if necessary, while preserving the pneumoperitoneum/pneumoretroperitoneum.

**Methods:**

Seventy-five consecutive donors receiving this procedure were retrospectively studied. A 2-cm skin incision was made at the midpoint between the tip of the 12th rib and superior border of the iliac bone in the midaxillary line, through which retroperitoneal space was made. Preperitoneal wound with a 6 – 7-cm pararectal incision in the upper abdominal region was connected to the retroperitoneal space. A GelPort was put inside the pararectal surgical wound. The principle was pure retroperitoneoscopic surgery; hand-assist was applied for retraction of the kidney in the renal vessel control and graft extraction.

**Results:**

The mean operation time including waiting time for recipient preparation was 242.2±37.0 (range: 214.0–409.0) min, and the mean amount of blood loss was 164.3±146.6 (range: 10.0–1020.0) ml. The mean WIT was 2.8±1.0 (range: 1.0–6.0) min. The shortage of renal vessels or ureter was observed in none of the grafts. No donor experienced blood transfusion, open conversion, or injury of other organs. Blood loss was greater in patients with body mass index (BMI) of 25 kg/m^2^ or higher than in those with BMI of <25 kg/m^2^ (218.4±98.8 vs. 154.8±152.1 ml, P=0.031). No donor had postoperative ileus or reported wound pain leading to decreased activity of daily life or wound cosmetic problem.

**Conclusions:**

Retroperitoneoscopic hand-assisted donor nephrectomy with the mentioned approach was suggested to be a feasible option without compromising safety, although further improvement in surgical techniques is warranted.

## Background

Laparoscopic donor nephrectomy has been developed to promote organ donation in living kidney transplantation, alleviating morbidities associated with conventional open surgery [1,2], and it is nowadays an accepted option supported by many studies reporting its excellent results with safety [3,4]. Donor nephrectomy with retroperitoneoscopic approaches has also been shown with encouraging perioperative and functional outcomes [5,6]. Compared to laparoscopic/transperitoneal donor nephrectomy, the retroperitoneoscopic technique potentially has advantages and disadvantages; it facilitates a direct hilar/vessel access and avoids mobilization of intraperitoneal organs, possibly leading to less incidence of postoperative ileus, whereas the limited working space and few anatomical landmarks represent shortcomings of the retroperitoneoscopic approach [7,8].

Wolf and associates reported the efficacy and safety of hand-assisted laparoscopic donor nephrectomy in 1998 [9,10], and modified methods have been preferred in the United States; 63.9% of donor nephrectomies were performed with hand-assisted laparoscopy in 2007 [4]. Finger exploration and dissection and immediate bleeding control with a direct pressure are conceivable benefits of hand-assist. In particular, the rapid extraction of the graft to preserve its function by shortening the warm ischemia time (WIT) is a major reason why hand-assisted approaches have been selected in donor nephrectomy [11]. To minimize morbidity and ensure safety in donors, we also have developed hand-assisted retroperitoneoscopic living donor nephrectomy, utilizing surgical devices such as hand-assist access port to enhance the aforementioned benefits. The present study was performed to assess the efficacy and safety of retroperitoneoscopic living donor nephrectomy with a gel-sealed hand-assist access device, GelPort, reporting perioperative and functional outcomes in patients/grafts receiving this technique.

## Methods

### Patients/donors

We reviewed the medical records of 75 consecutive patients/donors, who received hand-assisted retroperitoneoscopic living donor nephrectomy at Niigata University Hospital between October 2008 and March 2012. Written informed consent was obtained from all of them. The procedure for this research project and retrospective study was approved by the Ethics Committee of Niigata University. Renal vascular anatomy was evaluated using computed tomography. Donors with multiple renal arteries and/or veins were not excluded in the indication of this procedure; during the same period, an open nephrectomy was selected in a male marginal donor because of previous abdominal polysurgery and comorbidities. Patients’ demographics were shown in Table 1.

**Table 1:** Patients’ demographics (n=75)

	
Mean age (range) [y.o.]	55.7±10.4 (31–78)
Gender (male/female, n)	26/49
Mean height (range) [cm]	158.5±8.4 (142.0–182.0)
Mean weight (range) [Kg]	55.0±9.1 (40.8–80.5)
Mean BMI (range) [Kg/m^2^]	21.8±2.8 (15.9–29.6)
Graft side (left/right, n)	1/74

### Surgical procedures

The patient was placed in the standard right full-flank, mild lumbar flexion position. The surgeon and assistant stood on the back side of the patient. A 2-cm skin incision was made at the midpoint between the tip of the 12th rib and superior border of the iliac bone in the midaxillary line. Through this small wound, the lumbodorsal fascia was exposed, the fascia was bluntly detached from the muscular layer, and a retroperitoneal space was bluntly made. Thereafter, the retroperitoneal working space was extended by inflating a balloon dilator/dissector (PDB™ Sterile Balloon [Kidney Shape], Covidien, Mansfield, MA, USA) under the observation using an endoscope inserted through the balloon tip cannula; this wound was used as a scope port (12 mm blunt tip port). Subsequently, a 6 – 7-cm pararectal (border of the rectus abdominis) incision was made in the upper abdominal region, and in a pararectal approach, preperitoneal wound was made. This preperitoneal wound was connected to the mentioned retroperitoneal space. A hand-assist access device, GelPort, (Applied Medical, Rancho Santa Margarita, CA, USA) was put inside the pararectal surgical wound; the GelPort system is an innovative wound sealing device that permits the access of the hand to the surgical field, free trocar site choice and exchange within it, and rapid conversion to open surgery if necessary, while preserving the pneumoperitoneum/pneumoretroperitoneum [12].

**Figure 1:**
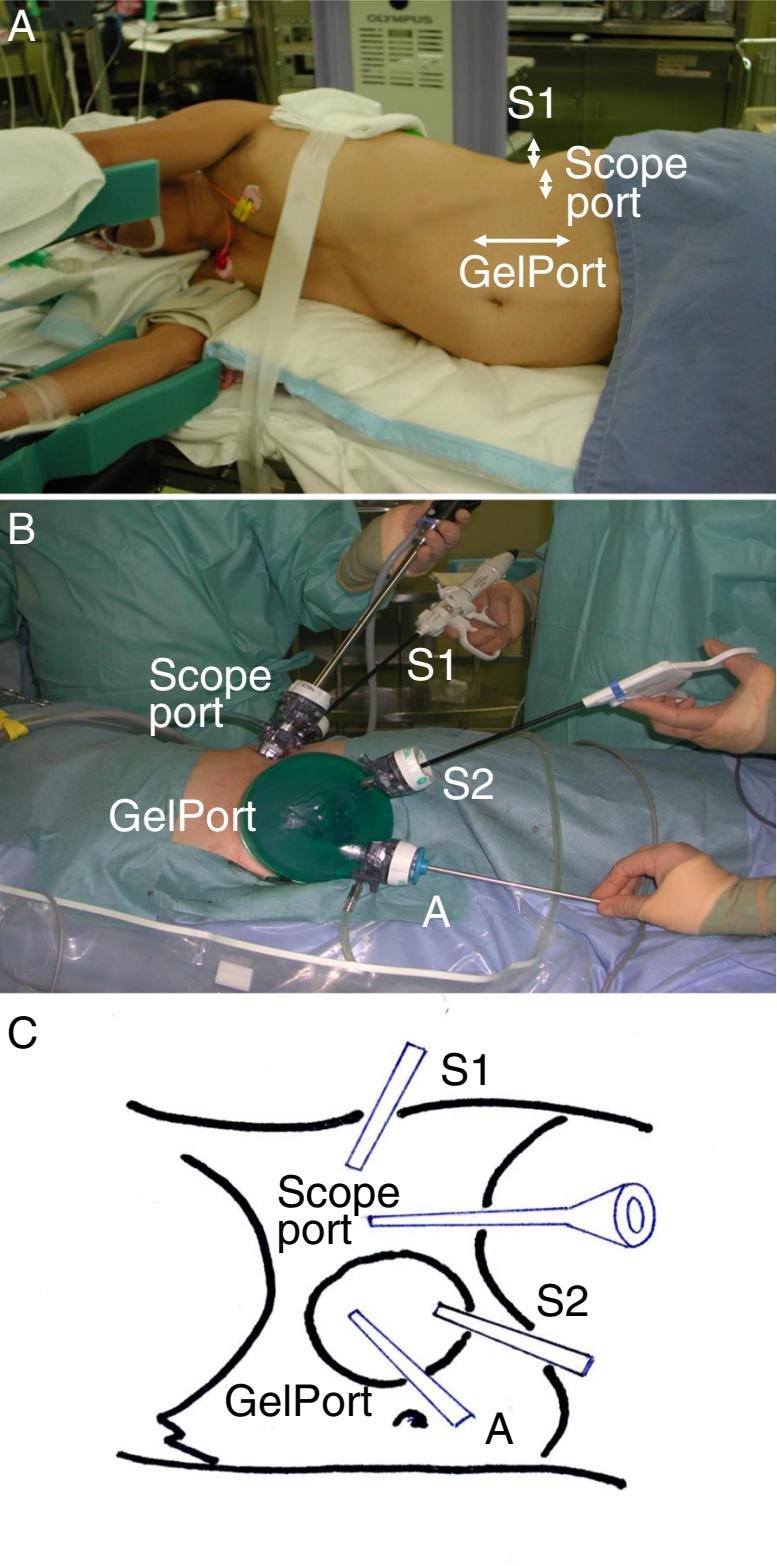
**A: Skin incisions. B**: The arrangement of GelPort, trocars and the scope port. The surgeon operated using the ports S1 (12-mm) and S2 (12-mm, through GelPort), and the port A (5-mm, through GelPort) was occasionally used by an assistant for retraction or suctioning. **C**: Schema.

The pneumoretroperitoneum was maintained by the insufflation of carbon dioxide at 10 – 12 mm Hg. Next, the 12-mm second port was placed between the 12th rib and superior border of the iliac bone on the posterior axillary line, and another 12-mm port and a 5-mm trocar were inserted penetrating GelPort. The arrangement of GelPort, trocars and the scope port was shown in Figure 1. The surgical procedures were in principle performed with the pure retroperitoneoscopic technique without hand-assist except for the control of renal vessels and graft extraction, and dissection of the renal upper pole and bleeding were occasionally managed with hand-assist. We first identified the proximal ureter. The Gerota's fascia/perirenal fat was dissected on the posterolateral surface. As the dissection was progressed, the pulsation of the renal artery was observed through the fat/connective tissue. Following the dissection of the renal artery and its mobilization near the bifurcation, the renal vein was identified and dissected; in left nephrectomy, ligation and transection of lumbar and adrenal veins were performed using titanium clips or vessel sealing systems (LigaSure vessel sealing system, Covidien, Boulder, Colorado, USA or EnSeal , Ethicon Endo-Surgery, OH, USA). The distal end of the gonadal vein was controlled similarly, and its proximal end was transected with sealing. For right nephrectomy, the renal artery was mobilized to the retrocaval site, and the renal vein was mobilized to the inferior vena cava. Hand-assisted retraction was occasionally applied to facilitate dissection of the upper pole.

**Figure 2:**
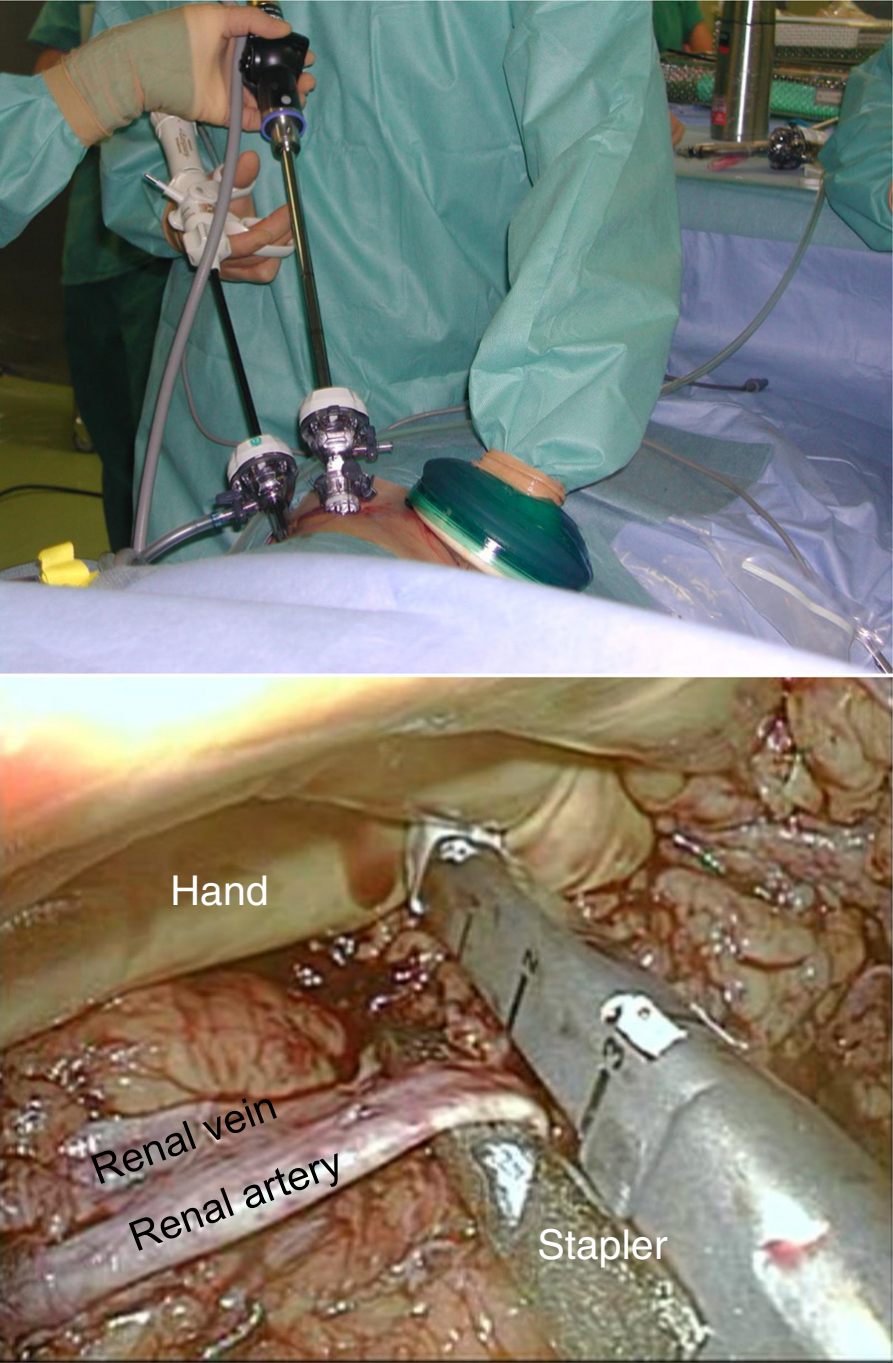
**In the control of renal vesels, the kidney was retracted by hand-assist to facilitate the access to the renal vessels.** Hand-assist through GelPort also enabled the rapid extraction of the graft.

Finally, the distal ureter was transected below the level of the iliac vessels. In the control of the renal vessels, to make a conspicuous surgical field and facilitate the access to them, the kidney was retracted by hand-assist. A linear non-cutting stapler (Autosuture Endo TA^TM^ 30 Covidien, Mansfield, MA, USA or Endocutter EZ45, Ethicon Endo-Surgery, OH, USA) was used to staple the artery, and the artery was transected with laparoscopic scissors, followed by a similar control for the renal vein and immediate graft extraction from GelPort (Figure 2).

### Statistical analysis

In addition to the chi-square test, Mann–Whitney U test was used to compare unpaired parameters between two subgroups. Correlations between parameters were analyzed using Spearman’s rank correlation coefficient (rs) analysis. Statistical analyses were calculated and tested using SPSS software version 15.0 (SPSS, Inc., Chicago, IL, USA) in Windows-based computers. The test was two-sided and p < 0.05 was considered significant.

## Results

### Perioperative outcomes

Perioperative outcomes were summarized in Table 2. The mean operation time was 242.2 ± 37.0 (range: 214.0 – 409.0) min; it included waiting time for differently-timed recipient preparation in 66 procedures. The mean amount of blood loss was 164.3 ± 146.6 (range: 10.0 – 1020.0) ml. The mean WIT was 2.8 ± 1.0 (range: 1.0 – 6.0) min, and the shortage of renal vessels or the ureter was observed in none of the patients. No patient experienced blood transfusion, conversion to open surgery, intraoperative injury of other organs, or postoperative bleeding. The intraoperative blood loss was positively correlated with operation time (Spearman’s rank correlation coefficient analysis, rs=0.275, p=0.018). The intraoperative blood loss was greater in patients with body mass index (BMI) of 25 kg/m^2^ or higher than in those with BMI of < 25 kg/m^2^ (218.4 ± 98.8 vs. 154.8 ± 152.1 ml, Mann–Whitney U test, p=0.031).

**Table 2:** Perioperative outcomes (n=75)

	
Operation time (range) [min]	242.2 ± 37.0 (214–409.0)
Blood loss(range) [ml]	164.3 ± 146.6 (10.0–1020.0)
WIT(range) [min]	2.8 ± 1.0 (1.0–6.0)
Open conversion (n)	0
Blood transfusion (n)	0
Injury of other organs (n)	0

### Short-term functional outcomes in donors and grafts

**Figure 3:**
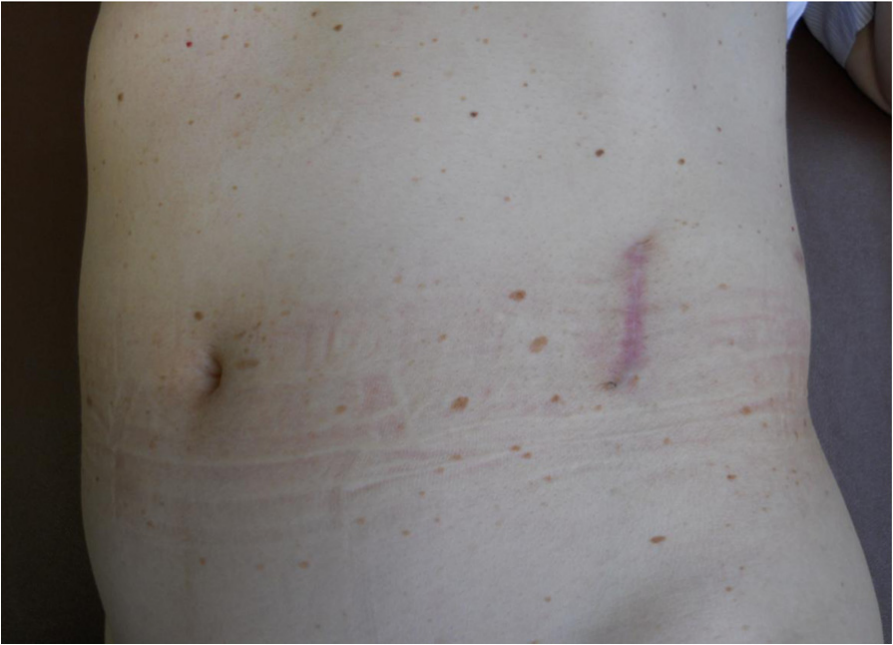
Postoperative appearance.

None of the patients developed surgical-site infection or ileus postoperatively (observation period: range 3 – 24, mean 12 months). No patients reported wound pain leading to decreased activity of daily life or cosmetic problems associated with the surgical wound (Figure 3). The mean serum creatinine level in recipients was 1.34 and 1.33 mg/dl one month and 3 months after surgery, respectively (Table 3). Additionally, donor-nephrectomy-related renal dysfunctions such as perfusion abnormality, perioperative ischemia, and thrombosis were absent in all of them.

**Table 3:** Graft function

	**1 month after surgery**	**3 months after surgery**
Mean serum creatinine (range) [mg/dL]	1.34±0.53	1.33±0.39
	(0.33–3.26)	(0.39–2.36)

## Discussion

The avoidance of surgery-related adverse events, minimizing WIT, and the prevention of graft dysfunction are rationales in living donor nephrectomy. Although transperitoneal/laparoscopic approaches are superior to retroperitoneoscopic surgery in acquiring a wide surgical field and anatomical orientation, postoperative ileus is possibly encountered in a fraction of donors treated transperitoneally [7,13]. In the present study, no postoperative ileus was observed, suggesting that the current retroperitoneoscopic approach is associated with less incidence of postoperative ileus.

We principally performed the procedures with the pure retroperitoneoscopic technique without hand-assist except for the control of renal vessels and graft extraction. In the present study, the mean WIT was 2.8 min; it was seemingly shorter than those in previous reports [11,14]. Although linear non-cutting stapler used in our patient series required additional disconnections/ transactions of the vessels, the hand-assisted retraction during the control and transection of the renal vessels facilitated the access to them and enabled prompt graft extraction from the wound.

Although it was previously reported that laparoscopic/retroperitoneoscopic donor nephrectomy under pneumoperitoneum/pneumoretroperitoneum was associated with reduced renal function possibly brought about by renal ischemia and/or perfusion abnormality [15], recent studies have shown that pneumoperitoneum does not appear to have an adverse impact on early graft reperfusion [16]. In our patient series, graft dysfunction involved in shortcomings of the surgical procedure or pneumoretroperitoneum was absent.

Laparoendoscopic single-site (LESS) surgery performed through a single small skin incision has been associated with less postoperative pain and fewer port site-related complications, and LESS-donor nephrectomy has recently been reported with similar perioperative outcomes and less adverse events represented by hernia, pain, and bleeding from epigastric vessel injury compared with those of laparoscopic donor nephrectomy [17]. LESS-donor nephrectomy is potentially superior to the current approach in minimization of the wound and cosmetic improvement, while instrument retraction to obtain surgical site exposure and manipulations of the kidney with multiple arteries may occasionally be limited in LESS-donor nephrectomy.

Several methods have been reported to develop hand-assisted retroperitoneoscopic donor nephrectomy. Wadström and associates reported a large case series; they performed 413 procedures and concluded that this technique reduces risk of intraabdominal complications [18]. They also utilized hand-assist devices such as LapDisc (Ethicon Endosurgery, Cincinnati, OH), placed in an inferior midline or Pfannenstiel incision in small donors. A blunt 12-mm working port and a 12-mm blunt port for a laparoscope were additionally placed, and hand-assist was applied in a positive manner throughout the procedure.

The present study had several limitations. It had a retrospective study design, and the study volume was not large. Also, the study did not have control arms, which could stress the advantage and disadvantage of the mentioned approach. Additionally, several points need to be improved for the applied procedure. The operation time was seemingly longer, although it included waiting time for differently-timed recipient preparation in 66 procedures (accurate time data missing). The injury of the diaphragm may potentially take place in the current approach [7], although this was absent in our patient series.

Short operation time and small intraoperative blood loss are favorable in donor nephrectomy. In our study, blood loss was positively correlated with the operation time, and also, BMI was an important parameter associated with blood loss. Larger volume of perirenal fat in donors with greater BMI may account for more frequent dissection/detachment procedures leading to greater blood loss.

## Conclusions

This is the initial report on retroperitoneoscopic/laparoscopic donor nephrectomy with a gel-sealed hand-assist access device, which is suggested to be a feasible and safe option. In combination with the endoscopic linear non-cutting stapler, this approach possibly contributes to the preservation of graft function and quality by curtailing the WIT. Further improvements in surgical techniques are warranted to minimize surgery-related morbidity and to maximize safety for living kidney donors.

## Abbreviations

WIT: Warm ischemia time; CT: Computed tomography; BMI: Body mass index; LESS: Laparoendoscopic single-site.

## Competing interests

The authors declare that they have no competing interests.

## Authors’ contributions

KA performed data collection and analysis, and assisted to draft the manuscript. TN participated in all surgical procedures in donors. NH wrote the manuscript and supervised throughout the study. KA, NH, TK, and KS performed surgical procedures in donors. KS and KT performed recipient surgery, and assisted to draft the manuscript. All authors read and approved the final manuscript.

## Pre-publication history

The pre-publication history for this paper can be accessed here:

http://www.biomedcentral.com/1471-2490/13/7/prepub
